# Supplementary material for: Zinc excess promotes lysosome remodeling by activating HLH-30/TFEB through the action of the high zinc sensor HIZR-1
Source: bioRxiv. 2026 Feb 24:2026.02.22.707197. Preprint. [Version 1] doi: 10.64898/2026.02.22.707197 (PMC13160143; doi:10.64898/2026.02.22.707197)

Figure S1

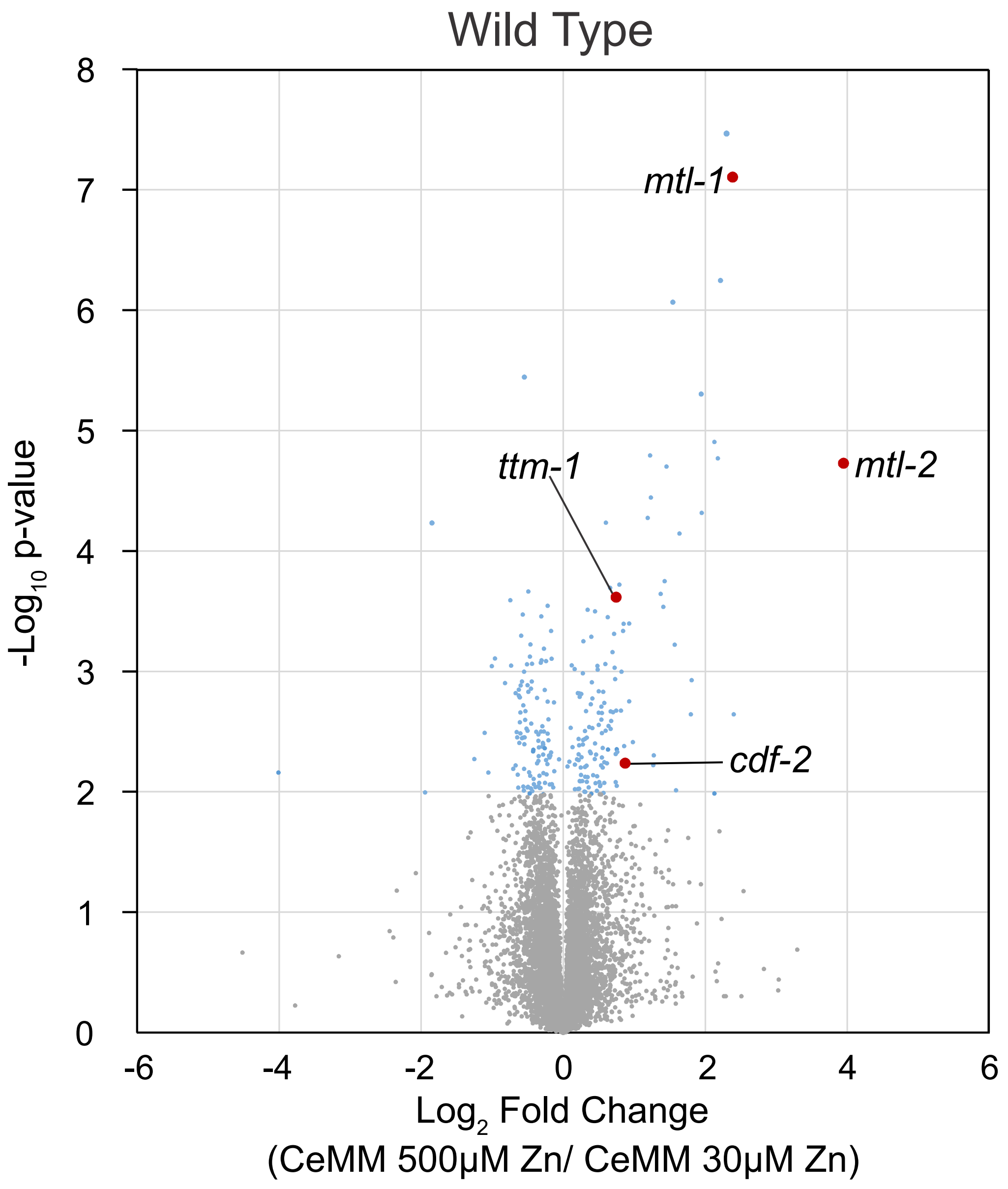

Wild Type
mtl-1
mtl-2
ttm-1
cdf-2
-Log10 p-value
Log2 Fold Change
(CeMM 500µM Zn/ CeMM 30µM Zn)
0
1
2
3
4
5
6
7
8
-6
-4
-2
0
2
4
6

Figure S2

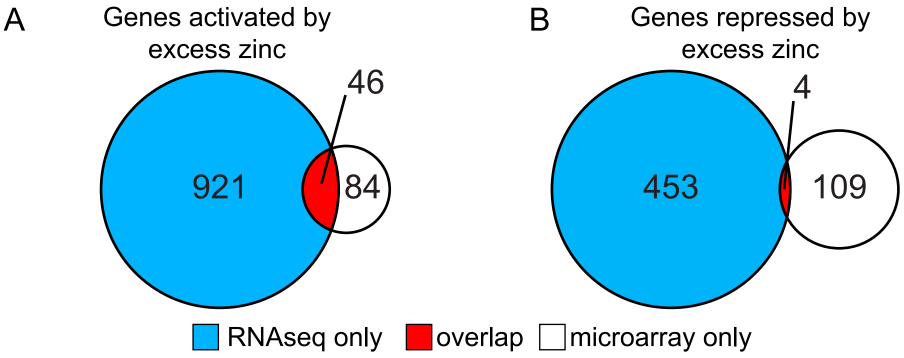

C

	RNAseq		Microarray		qPCR	
Gene	Log_2 Fold Change	*p*-value	Log_2 Fold Change	*p*-value	Log_2 Fold Change	*p*-value
asp-17	6.95	7.34E-14	2.22	5.67E-07	7.50	<0.0001
mtl-1	6.92	3.20E-27	2.39	7.89E-08	7.16	<0.0001
gst-33	5.83	1.99E-15	1.81	1.18E-03	5.07	<0.0001
mtl-2	4.45	8.73E-21	3.95	1.87E-05	6.46	<0.0001
cdf-2	3.91	2.34E-21	0.87	5.81E-03	5.80	<0.0001
B0024.4	3.82	2.25E-07	1.43	1.79E-04	N.D.	
cpr-1	3.63	5.53E-11	1.37	2.27E-04	3.80	0.0002
thn-2	3.4	1.04E-18	1.22	1.61E-05	3.50	<0.0001
spp-11	3.33	6.56E-04	1.57	6.03E-04	N.D.	
F54B8.4	3.08	1.11E-13	0.33	8.29E-03	N.D.	
ttm-1	3.07	1.49E-21	0.75	2.43E-04	3.73	<0.0001
B0507.8	3.02	2.62E-07	0.75	4.44E-03	N.D.	
T01D3.6	2.62	2.32E-07	0.72	9.35E-04	N.D.	
lro-1	2.57	1.15E-08	0.45	3.17E-04	N.D.	
srlf-35	2.57	1.34E-11	0.56	1.48E-03	N.D.	
cyp-33C5	2.48	4.73E-08	0.17	9.55E-03	N.D.	
lys-7	2.43	5.41E-06	0.55	6.30E-03	N.D.	
C29F3.7	2.26	3.05E-18	1.54	8.60E-07	N.D.	
K05B2.4	2.23	2.29E-09	0.67	2.16E-03	N.D.	
C29F7.2	2.17	2.54E-17	0.73	1.16E-03	2.82	<0.0001
W07B8.4	2.07	2.86E-04	0.93	4.02E-04	N.D.	
F53A9.1	2.04	7.71E-06	0.23	1.53E-03	N.D.	
clec-52	1.81	1.84E-06	1.45	1.99E-05	4.79	<0.0001
cyp-33C4	1.77	1.72E-07	0.6	5.84E-05	2.74	<0.0001
K08D8.6	1.56	1.76E-13	0.59	8.68E-04	N.D.	
ncx-7	1.49	2.46E-10	0.98	3.87E-03	2.04	<0.0001
R05H10.1	1.33	3.41E-04	0.56	4.32E-03	N.D.	
Y46G5A.20	1.31	5.86E-04	0.37	2.91E-03	N.D.	
R09F10.1	1.23	9.31E-10	0.28	4.05E-03	N.D.	
T07D3.4	1.19	3.76E-06	0.7	2.18E-03	N.D.	
npa-1	1.17	4.23E-06	0.39	4.79E-03	1.77	<0.0001
tth-1	1.13	3.82E-08	0.16	9.59E-04	N.D.	
T21H3.1	1.1	1.15E-12	0.6	3.11E-03	N.D.	
clec-83	1.07	2.46E-07	0.34	3.08E-04	N.D.	
ugt-51	1.01	2.07E-04	0.82	1.01E-03	N.D.	
clec-66	0.99	3.27E-08	0.79	1.91E-04	N.D.	
apy-1	0.98	1.32E-09	0.54	1.96E-03	N.D.	
lys-1	0.97	5.91E-10	0.5	2.21E-03	N.D.	
lys-2	0.96	1.20E-08	0.26	1.54E-03	N.D.	
Y43C5A.3	0.93	1.57E-05	0.39	1.88E-03	N.D.	
asm-2	0.87	1.98E-05	1.19	5.31E-05	N.D.	
scl-5	0.85	4.58E-04	0.33	6.09E-03	N.D.	
tmem-135	0.83	8.84E-06	0.81	2.12E-03	N.D.	
T02C5.1	0.69	4.29E-06	0.33	3.56E-03	N.D.	
ctsa-1.2	0.62	1.24E-06	0.36	9.19E-03	N.D.	
W01A11.1	0.47	9.39E-04	0.31	3.12E-03	N.D.	
acp-6	-0.55	1.37E-05	-0.31	3.17E-03	N.D.	
F44E7.5	-0.63	5.75E-05	-0.61	3.27E-03	N.D.	
C55B7.3	-0.88	4.35E-04	-1.25	5.34E-03	N.D.	
C14F11.4	-0.88	3.61E-04	-0.56	1.91E-03	N.D.	

Figure S3

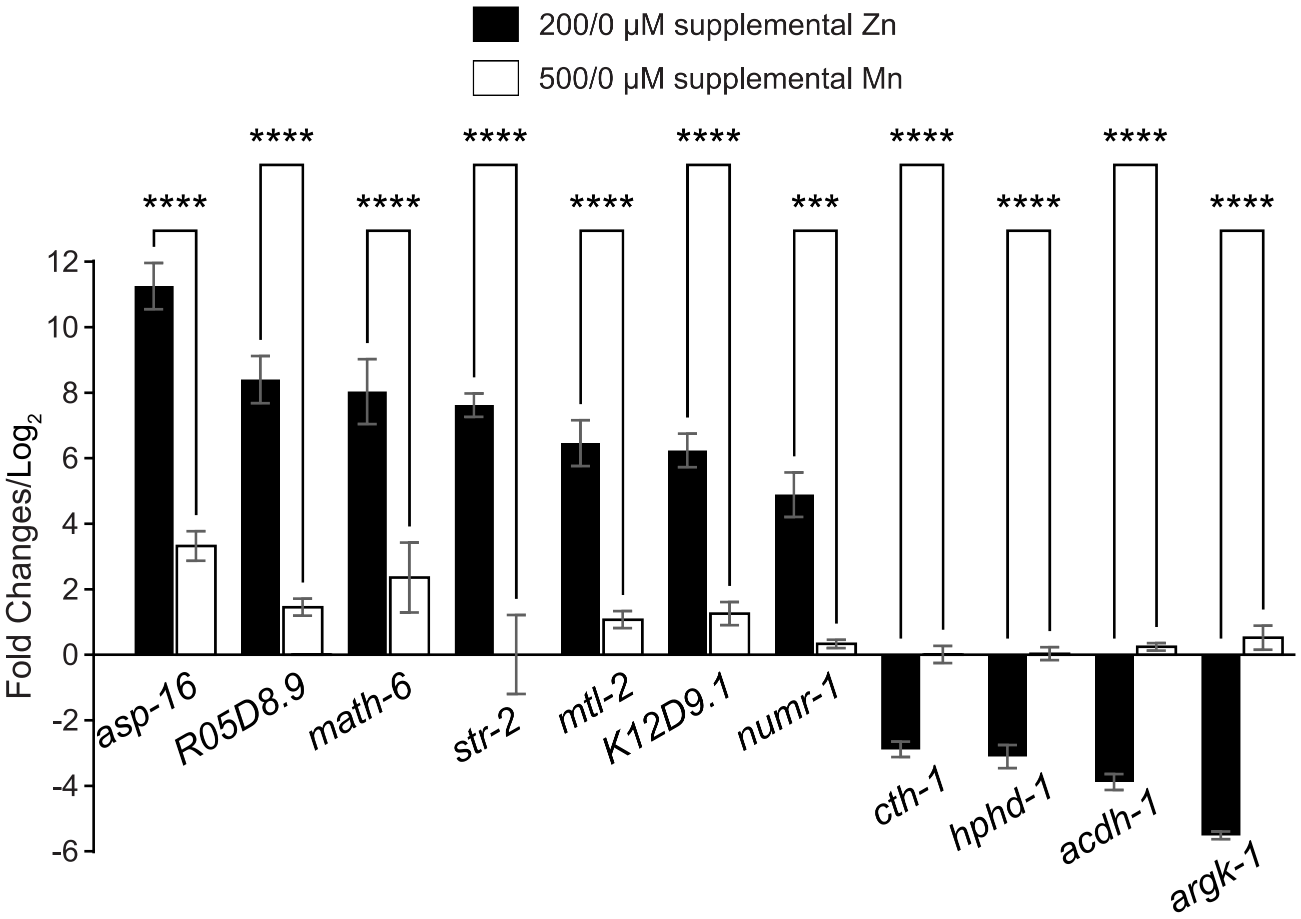

200/0 µM supplemental Zn
500/0 µM supplemental Mn
Fold Changes/Log2
12
10
8
6
4
2
0
-2
-4
-6
****
****
****
****
****
****
***
****
****
****
****
asp-16
R05D8.9
math-6
str-2
mtl-2
K12D9.1
numr-1
cth-1
hphd-1
acdh-1
argk-1

Figure S4

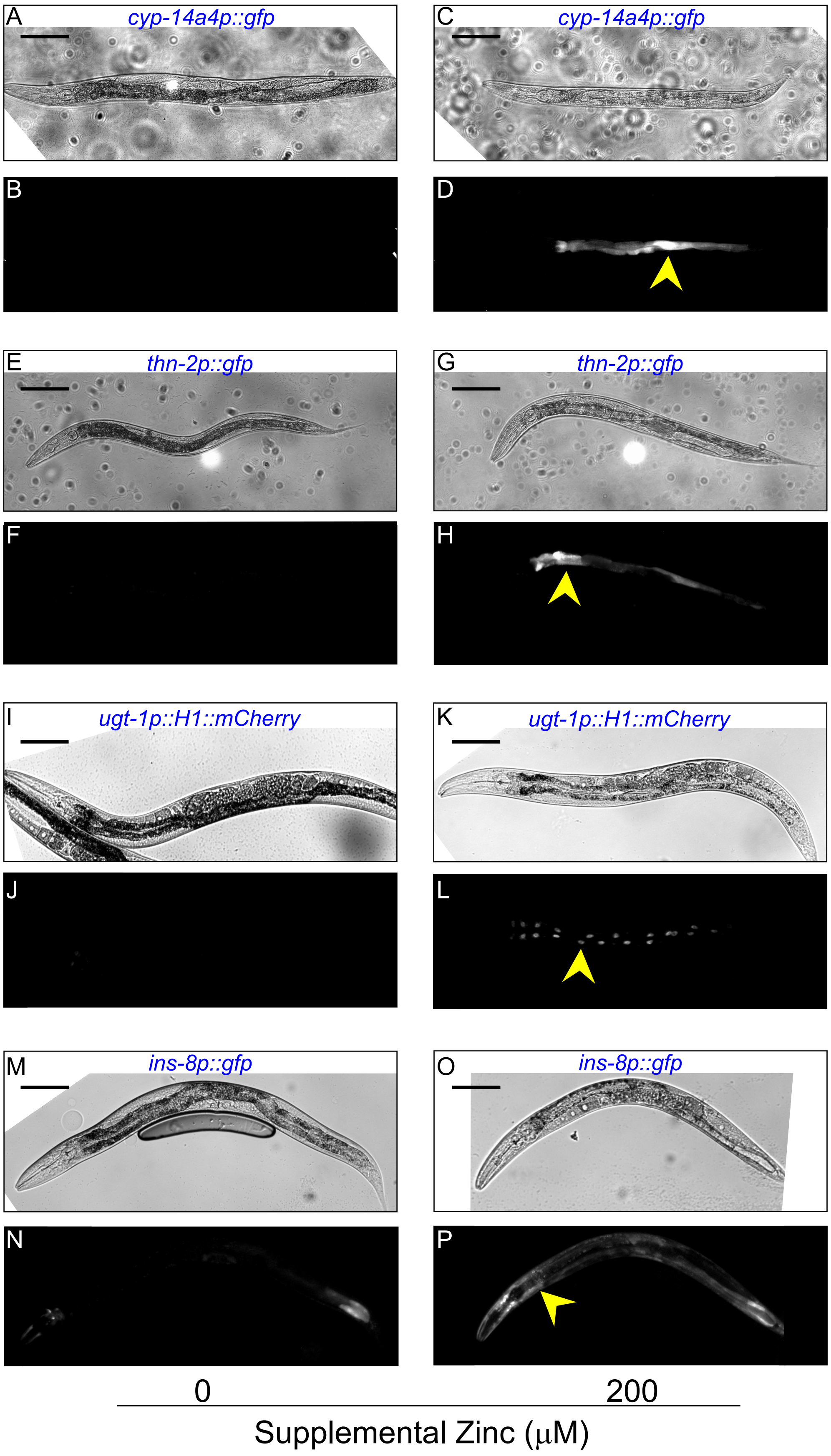
A
cyp-14a4p::gfp
C
cyp-14a4p::gfp
B
D
E
thn-2p::gfp
G
thn-2p::gfp
F
H
I
ugt-1p::H1::mCherry
K
ugt-1p::H1::mCherry
J
L
M
ins-8p::gfp
O
ins-8p::gfp
N
P
0
200
Supplemental Zinc (μM)

Figure S5

A

Gene	Position	E-box Sequence	*p*-value
hlh-30	-1165	ACACGCGA	6.60E-04
hizr-1	**-563**	A**CACG**G**G**A	2.40E 04
lipl-1	-439	CCACGTGG	5.90E-04
lipl-2	-55	ACACGTGT	9.00E-05
lipl-3	-431	ACACGTGC	4.50E-04
sqst-3	-175	TCACGTGG	3.10E-05

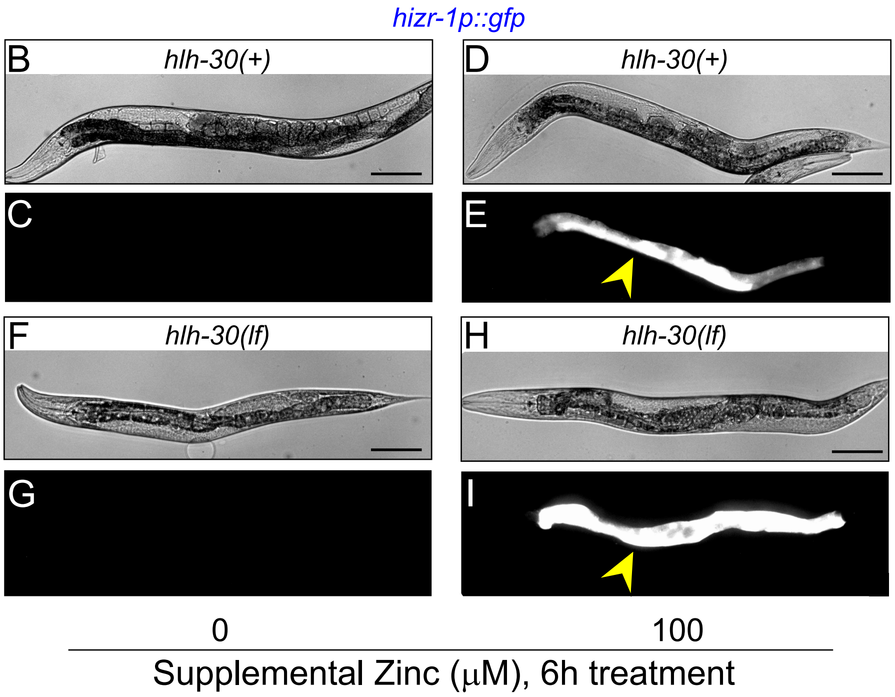

Supplement: Supplement 1 — Supplemental Figure 1 (with main Figure 1). Identification of zinc-regulated genes by microarray analysis. Populations of WT worms were cultured in fully defined CeMM medium containing 30μM ZnCl2 for several weeks (Davis et al. 2009). To analyze transcription in zinc excess conditions, worms were transferred to CeMM containing 500μM ZnCl2 and cultured for 6 days. A synchronous population of L4 staged worms was obtained using a COPAS-BIOSORT. Four biological replicates were performed for 30μM and 500μM ZnCl2 CeMM. RNA was isolated and analyzed using microarrays containing ~19,000 C. elegans genes. Each point is data for one gene, showing Log2 fold change of expression level and the P value. Blue and gray indicate significant and nonsignificant P values, respectively. Red indicates genes previously established to be zinc-activated. Supplemental Figure 2 (with main Figure 1). Comparison of zinc-regulated genes identified by RNA seq and microarray analysis. (A,B) Venn diagrams show the overlap (red) between lists of genes identified by RNA seq (blue circle) and microarray (white circle). Panel A displays genes activated by excess zinc, and panel B displays genes repressed by excess zinc. For activated genes, ~35% of the 130 genes identified by microarray were also identified by RNA seq. For repressed genes, ~4% of the 113 genes identified by microarray were also identified by RNA seq. (C) A list of the overlapping genes with the Log2 Fold Change and p-value for the method of RNA seq and microarray. Fourteen of these activated genes were analyzed by qRT-PCR, and all displayed activation by this method. Supplemental Figure 3 (with main Figure 1). Excess manganese did not cause the same changes in gene expression as excess zinc. Mixed-stage populations of WT worms were cultured on standard (replete for zinc and manganese), zinc excess (200μM supplemental zinc), or manganese excess (500μM supplemental manganese) NAMM dishes for 16–18 hours, and gene expression was analyzed [file media-1.pdf]
